# Cellular senescence of renal tubular epithelial cells in acute kidney injury

**DOI:** 10.1038/s41420-024-01831-9

**Published:** 2024-02-05

**Authors:** Juan Chen, Huhai Zhang, Xiangling Yi, Qian Dou, Xin Yang, Yani He, Jia Chen, Kehong Chen

**Affiliations:** 1 Department of Nephrology, Daping Hospital, Army Medical University, 400042 Chongqing, China; 2 Department of Nephrology, Southwest Hospital, Army Medical University, 400042 Chongqing, China; 3 State Key Laboratory of Trauma, Burn and Combined Injury, Army Medical University, Chongqing, China

**Keywords:** Ageing, Kidney

## Abstract

Cellular senescence represents an irreversible state of cell-cycle arrest during which cells secrete senescence-associated secretory phenotypes, including inflammatory factors and chemokines. Additionally, these cells exhibit an apoptotic resistance phenotype. Cellular senescence serves a pivotal role not only in embryonic development, tissue regeneration, and tumor suppression but also in the pathogenesis of age-related degenerative diseases, malignancies, metabolic diseases, and kidney diseases. The senescence of renal tubular epithelial cells (RTEC) constitutes a critical cellular event in the progression of acute kidney injury (AKI). RTEC senescence inhibits renal regeneration and repair processes and, concurrently, promotes the transition of AKI to chronic kidney disease via the senescence-associated secretory phenotype. The mechanisms underlying cellular senescence are multifaceted and include telomere shortening or damage, DNA damage, mitochondrial autophagy deficiency, cellular metabolic disorders, endoplasmic reticulum stress, and epigenetic regulation. Strategies aimed at inhibiting RTEC senescence, targeting the clearance of senescent RTEC, or promoting the apoptosis of senescent RTEC hold promise for enhancing the renal prognosis of AKI. This review primarily focuses on the characteristics and mechanisms of RTEC senescence, and the impact of intervening RTEC senescence on the prognosis of AKI, aiming to provide a foundation for understanding the pathogenesis and providing potentially effective approaches for AKI treatment.

## Facts

1. Cellular senescence represents an irreversible state of cell-cycle arrest, which has senescence-associated secretory phenotype (SASP) and apoptotic resistance phenotype.
2. Renal tubular epithelial cells (RTEC) senescence is a critical cellular event in the progression of acute kidney injury (AKI), which promotes the transition of AKI to chronic kidney disease.
3. Inhibiting RTEC senescence, targeting the clearance of senescent RTEC, or promoting the apoptosis of senescent RTEC hold promise for enhancing the renal prognosis of AKI.

## Open questions

1. What are the differences in the characteristics and phenotypes of senescent renal tubular epithelial cells (RTEC) in distinct stressors-induced AKI?
2. What is the heterogeneity of the role and mechanism of senescent RTEC in distinct stressors-induced AKI?
3. How to improve the targeting and effectiveness of intervening RTEC senescence to further ameliorate the prognosis of AKI?

## Introduction

Acute kidney injury (AKI) is a prevalent and life-threatening clinical condition characterized by a rapid decline in kidney function, with a high incidence and mortality rate [1]. However, the prognosis for surviving patients with AKI is far from optimistic. Research has shown that 30% to 70% of AKI survivors progress to chronic kidney disease (CKD), and approximately 17% of patients develop end-stage renal disease (ESRD) within one year, necessitating renal replacement therapy. This places a substantial economic burden on both society and affected families [2]. The causes of AKI encompass sepsis, nephrotoxic drugs, ischemia-reperfusion injury, and contrast-induced nephropathy. Regrettably, effective treatment strategies for AKI are currently lacking. Therefore, it is imperative to comprehensively elucidate the mechanisms underlying AKI for its prevention and treatment.

Renal tubular epithelial cells (RTECs) stand as among the most critical cells in the renal parenchyma, significantly influencing the physiological functions of the kidneys. These cells are characterized by their abundant mitochondria, signifying their high metabolic and energy demands. Situated in areas of the kidney with limited blood flow and oxygen supply, RTECs are inherently more vulnerable to damage. Additionally, they are predisposed to the accumulation and concentration of various harmful substances, rendering them the primary target cells of AKI [3]. Numerous studies have demonstrated that RTECs undergo necrosis, apoptosis, and ferroptosis following AKI. Residual RTECs possess robust proliferative and regenerative capacities that influence the prognosis of AKI. In mild and moderate AKI, RTECs quickly re-enter the cell cycle from a quiescent state and undergo proliferation and regeneration, thereby promoting the restoration of kidney structure and function. However, in severe AKI, the regenerative capacity of the residual RTEC is limited, leading to abnormal repair. Moreover, RTECs are prone to mitochondrial dysfunction, oxidative stress, and disturbances in energy metabolism, resulting in stress-induced cellular senescence. RTEC senescence not only hampers renal repair but also triggers the secretion of a multitude of senescence-associated secretory phenotypes (SASP), such as inflammatory factors and chemokines, which accelerate the progression of AKI to CKD. RTEC senescence, an early cellular event, plays a pivotal role in the development of AKI. Evidence suggests that RTEC senescence is associated with a decrease in renal recovery rate. This review primarily focuses on the characteristics and effects of cellular senescence, the mechanisms of RTEC senescence in AKI, and the impact of intervening RTEC senescence on the prognosis of AKI, aiming to provide a foundation for understanding the pathogenesis and treatment of AKI.

## Cellular senescence

### Characteristics and phenotypes of cellular senescence

Cellular senescence can be classified into two primary types: replicative and stress-induced premature senescence [4]. Replicative senescence primarily occurs due to telomere shortening and a decrease in telomerase activity, eventually leading to reaching the Hayflick limit [5]. Stress-induced premature senescence is independent of chronological aging and is initiated by various pathological triggers, such as DNA damage, oxidative stress, and oncogenes. Unlike quiescent cells, senescent cells experience an irreversible cell-cycle arrest at either the G1/S or G2/M phase and are incapable of proliferating or differentiating, even when subjected to specific stimuli.

**Fig. 1:**
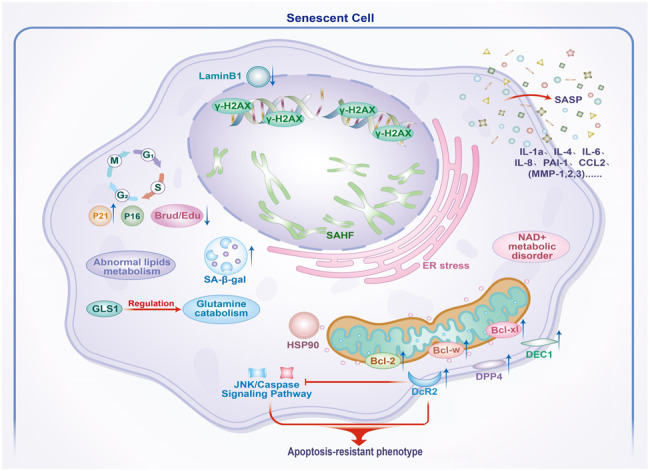
The phenotypes and markers of cellular senescence. Cellular senescence is an irreversible state of cell-cycle arrest, which exhibits SASP and apoptotic resistance phenotypes. Senescent cells are enlarged and have an irregular shape. The phosphorylation of γH2AX increases following DNA damage in cellular senescence. SAHF accumulates and Lamin B1 downregulates in the nucleus of the senescent cell. Enhanced lysosomal content (SA-β-gal), ER stress, and mitochondrial dysfunction are observed in senescent cells. Moreover, lipid metabolism, glutamine catabolism, and NAD+ metabolism are abnormal after cellular senescence.

Senescent cells exhibit several morphological alterations, including cellular enlargement, flattening, darker nuclear staining, and chromatin aggregation. Additionally, these cells secrete SASP and display resistance to apoptosis (Fig. 1). Recent studies employing ATAC-seq, RNA-seq, and ChIP-seq have identified NAT1, PBX1, and RRM2 as potential novel biomarkers of senescence and senescence-related diseases [4].

#### Cell morphology

Cellular senescence is characterized by irregular cell shape and cell enlargement, exceeding twice the size of normal cells, a hallmark feature of senescent cells [6]. Alterations in nuclear morphology and structure are signs of senescence, featuring enlarged multinucleated cells. The loss of nuclear membrane protein B1 (Lamin B1) contributes to nuclear membrane breakdown and the disruption of nuclear integrity, leading to condensed chromatin, the formation of heterochromatic foci, and the appearance of chromatin fragments in the cytoplasm, referred to as cytoplasmic chromatin fragments (CCFs) [7]. Aberrations in cell nucleus morphology and mutations in nuclear proteins such as MAN1, Nesprin-1, Emerin, and Torsin1, caused by premature senescence, are associated with various diseases, including cancer [8]. Additionally, cell organelles exhibit morphological abnormalities, characterized by an increased number of lysosomes and elevated β-galactosidase activity. Mitochondrial swelling and dysfunction result in the generation of excessive reactive oxygen species (ROS) [9].

#### Cell-cycle arrest

Cell-cycle regulation primarily relies on cell-cycle checkpoints, including the G1/S, G2/M, and spindle checkpoints. The cell cycle is influenced by various factors, such as cyclins, cyclin-dependent kinases (CDKs), and cyclin-dependent kinase inhibitors (CKIs). Cyclins form complexes with CDKs, leading to the phosphorylation of the retinoblastoma protein (RB) and the subsequent release of E2F, which facilitates the cell-cycle transition from the G1 to S phase.

Cell-cycle arrest is a primary characteristic of cellular senescence. Disruption in the regulation of cell-cycle progression can lead to cellular senescence. Unlike quiescent and terminally differentiated cells, senescence-related cycle arrest is irreversible, and senescent cells do not re-enter the cell cycle in response to any known physiological stimuli [7]. Cell-cycle arrest during the G1/S and G2/M phases induces cellular senescence [10, 11]. CKIs play a crucial role in the regulation of cellular senescence by promoting cell-cycle arrest. There are two principal families of CKIs: the Ink4 family and the KIP family. The Ink4 family includes p15, p16, p18, and p19, which inhibit the activity of CDK4/CDK6. Meanwhile, members of the KIP family, such as p21 and p27, reduce the activity of cyclin-CDK complexes, thereby promoting cell-cycle arrest [12]. The main factors driving cell-cycle arrest are CDKN2A (p16INK4, abbreviated as p16) and CKDN1A (p21CIP, abbreviated as p21). Both p21 and p16 inhibit the activity of CDK, leading to the non-phosphorylation of RB. This non-phosphorylated RB blocks the cell-cycle transition from the G1 to S phase [11]. Cell-cycle arrest during cellular senescence is primarily regulated by the p16INK4a/RB and p53/p21CIP1 pathways. Additionally, p53 induces cell-cycle arrest either at the G1/S or G2/M checkpoint, playing a pivotal role in the regulation of cellular senescence [13]. Post-translational modifications, such as ubiquitination, phosphorylation, acetylation, and SUMOylation, play crucial roles in regulating the levels and activity of p53.

#### Senescence-associated secretory phenotype (SASP)

Senescent cells exhibit metabolic activity and persistently secrete inflammatory factors, chemokines, matrix metalloproteinases, and other components of the SASP. The SASP fosters the senescence of neighboring cells and establishes a tissue microenvironment through autocrine and paracrine signaling. The composition and levels of the SASP vary based on the duration of senescence, senescence-induced stress, and cell type [14]. Furthermore, the composition and effects of the SASP diverge in different organs and disease types. In cases of skin trauma, the primary SASP components include CYR61 and PDGF-AA, which participate in the repair process. Tumor-induced senescence in liver cells is characterized by CCL-2, inducing immune surveillance effects [15]. In cases of aging-induced chronic senescence, IL-6, and PAI-1 contribute to chronic tissue damage and organ dysfunction [16], In CKD, the SASP predominantly consists of IL-1, 4, 6, 18, TGF-β, TNF-α, MMP-2, and PAI-1, which contribute to renal inflammation and fibrosis, thereby accelerating the deterioration of renal function [17]. In addition, SASP activation is regulated by transcription factors and epigenetic mechanisms.

#### Apoptosis resistance

Senescent cells generate various anti-apoptotic molecules that prevent their recognition and clearance by immune cells. The accumulation of senescent cells and the SASP exacerbate the progression of diseases [18, 19]. Multiple biological pathways, such as Bcl-2/Bcl-XL, p53/p21, and PI3K/AKT, are involved in the apoptotic resistance of senescent cells [20]. Both mRNA and protein levels of Bcl-W and Bcl-xL are upregulated in senescent cells, highlighting the pivotal role of the BCL-2 protein family in apoptosis resistance among senescent cells [21, 22]. Recent studies have revealed that the decoy receptor DcR2 is upregulated in senescent cells, and it functions to inhibit apoptosis in senescent fibroblasts and RTECs [23, 24]. Furthermore, p21 can inhibit apoptosis in senescent cells by suppressing the JNK and caspase signaling pathways [25]. The heat shock protein HSP90 emerges as a crucial factor for the survival of senescent cells, and the application of HSP90 inhibitors selectively induces apoptosis in senescent cells [20]. In a recent study, glutaminase 1 (GLS1) was found to inhibit senescent cell apoptosis by regulating glutamine metabolism [26].

### Physiological functions of cellular senescence

Cellular senescence serves diverse roles in various species. It has proven beneficial in mitigating pathological stress during the physiological and developmental processes of fish, amphibians, and mammals. Notably, cellular senescence not only regulates tissue remodeling in the course of embryonic development but also contributes to processes such as regeneration, repair, and tumor suppression [27].

#### Embryonic development

Cellular senescence constitutes a highly conserved and regulated process that unfolds during embryonic development. It actively participates in tissue remodeling, development, and morphogenesis, which are developmental processes featuring programmed senescence events [28, 29]. Senescent cells are detectable in various tissues and organs, especially in the kidney [30], inner ear [31], limbs, and nervous system, throughout embryonic development. The senescence marker p21 has been identified in the early stages of embryonic development, and its knockout results in abnormal kidney development, indicating that p21 plays a key role in embryonic development. Furthermore, p21 is not synchronized with other senescent markers such as p16 and p53, and it is regulated by the TGF-β/SMAD and P13K/FOXO pathways [28]. Cellular senescence is further associated with transient structures, such as the pronephros in salamanders and the yolk sac in zebrafish [5], both of which are contingent upon WNT and Hedgehog pathways. However, in amphibians, senescence primarily relies on the TGF-β signaling.

#### Regeneration and repair

Acute senescence plays a crucial physiological role in wound healing and tissue homeostasis [32]. During the early stages of trauma repair, senescent cells stimulate myofibroblast differentiation, facilitating scar formation and wound healing through paracrine signaling mechanisms, such as via platelet-derived growth factor AA (PDGF-AA). However, the removal of senescent cells results in delayed wound repair [33]. In the latter stages of trauma repair, senescent fibroblasts secrete antifibrotic factors that inhibit tissue fibrosis, thereby supporting adaptive repair processes [34]. Senescent fibroblasts and endothelial cells promote the healing of compromised skin, while the elimination of senescent cells yields opposing effects [33]. Similar roles for senescent fibroblasts have been reported in the context of myocardial infarction [35]. Moreover, senescent hepatic stellate cells decrease the secretion of extracellular matrix and enhance the production of extracellular matrix-degrading enzymes in liver injury models, thereby enhancing immune surveillance and restraining liver fibrosis [36]. In the early stages of ischemia-reperfusion-induced AKI, stress-induced senescent RTECs resist necrosis and apoptosis, consequently promoting the proliferation and regeneration of other RTECs [37]. This underscores the vital role of cellular senescence in the regeneration and repair of diverse organs following injury.

#### Tumor suppression

Numerous studies have shown that cellular senescence plays an important role in tumor suppression. The mechanisms underlying cellular senescence in the context of cancer primarily encompass oncogene-induced senescence (OIS), therapy-induced senescence (TIS), and the loss of tumor suppressor genes, exemplified by PTEN loss-induced cellular senescence (PICS) [38]. The p53 pathway plays a central role in both OIS and PICS. In OIS, the activation of oncogenes leads to DNA damage, subsequently activating p53 and leading to cellular senescence. In the case of PICS, p53 signaling is triggered through the mTOR pathway, even in the absence of DNA damage. Senescent cells can induce the senescence of neighboring tumor cells via the SASP, thereby inhibiting tumor progression. Remarkably, there exists a positive correlation between the low expression of senescence markers and a poor prognosis in renal cancer. The selective inhibition of cellular senescence leads to accelerated tumor progression, underscoring the protective effect of cell senescence in renal tumors. In addition, the inflammatory factors and chemokines secreted by tumor-induced senescent liver cells promote the chemotaxis of immune cells, thereby facilitating the elimination of tumor cells. Conversely, the elimination of senescent liver cells accelerates the progression of liver tumors, demonstrating that cellular senescence actively promotes and enhances the function of immune cells in clearing tumor cells.

### Pathological roles of cellular senescence

Extensive evidence substantiates that senescent cells initiate the onset of diseases and accelerate the progression of various diseases [39, 40]. Cellular senescence is not only associated with age-related degenerative diseases but also plays a key role in the pathogenesis of tumors, metabolic diseases, and acute as well as chronic kidney diseases. Senescent cells, along with the SASP, represent fundamental mechanisms that drive or exacerbate disease progression. In addition, senescent cells demonstrate resistance to apoptosis, which consequently results in the accumulation of senescent cells and SASP, thereby intensifying tissue damage and dysfunction.

#### Neurodegenerative diseases

A large accumulation of senescent cells is evident in brain tissue associated with neurodegenerative diseases, most notably Alzheimer’s and Parkinson’s disease. Senescent astrocytes have been directly linked to the onset of these diseases, while tau protein accumulation is closely correlated with brain aging [41]. In this sense, the elimination of senescent glial cells can prevent cognitive decline caused by the accumulation of tau protein [42, 43]. These findings suggest that cellular senescence plays a crucial role in the occurrence and progression of neurodegenerative diseases.

#### Tumor pathogenesis

Beyond its protective effects on tumors, cellular senescence also exerts adverse impacts on tumorigenesis. Increasing evidence suggests that senescence, in conjunction with the senescent microenvironment, is associated with cancer development and metastasis [44–46]. Senescent cells secrete MMP3, which in turn promotes tumor cell invasion and angiogenesis [47]. Furthermore, the SASP facilitates the transition from epithelial to mesenchymal cells, a transformation involved in the progression of metastatic cancer [48]. SASP constituents secreted by malignant cells promote immune evasion and drug resistance in tumor cells [49]. Moreover, senescence-related genes hold significant clinical relevance in the prognosis and immunotherapy of patients with cancer. For instance, the specific clearance of p16-positive cells retards tumor progression and diminishes tumor metastasis [50].

#### Diabetes

Senescent cells release a large number of SASP factors, which contribute to dysfunction in pancreatic beta cells and adipose tissue, thereby amplifying insulin resistance in peripheral tissues and culminating in the development of type 2 diabetes. Metabolic abnormalities, such as high blood sugar, the presence of its byproducts, and advanced glycation end products (AGEs), foster cellular senescence, thus further fueling the progression of diabetes [51]. In diabetes mouse models induced by high glucose, pancreatic beta cell proliferation is decreased, while the expression of senescence markers is increased [52, 53]. In addition, individuals with diabetes exhibit an increased susceptibility to age-related conditions, such as renal dysfunction, Alzheimer’s disease, cardiovascular disease, and visual impairment. Numerous studies have confirmed that a large number of senescent cells can be found in diabetic nephropathy, diabetic retinopathy, and vascular lesions stemming from diabetes. These senescent cells promote dysfunction, yet the inhibition of cellular senescence significantly ameliorates these deleterious effects. These findings suggest the pivotal role of cellular senescence in the progression of diabetes and its associated complications.

#### Kidney diseases

The kidneys are among the organs in the body that age most rapidly. In recent years, the role of renal parenchymal cell senescence in kidney disease has gradually gained attention. Various pathogenic factors stimulate the senescence of renal parenchymal cells, including tubular, mesangial, endothelial, and other cell types. Numerous studies have confirmed that senescence of renal parenchymal cells is a pivotal pathological feature in conditions such as diabetic nephropathy (DN), IgA nephropathy (IgAN), hypertensive nephropathy, and other CKD. It plays a substantial role in the progression of CKD [54]. Consequently, CKD is considered as a clinical model of renal senescence. Cellular senescence is closely linked to glomerulosclerosis, tubular atrophy, interstitial fibrosis, and the progression of DN in mouse models of diabetes induced by streptozotocin [55, 56]. In the context of DN, the inhibition of RTEC senescence significantly alleviates kidney damage and preserves renal function, thus delaying the progression of DN [57, 58]. Among patients with IgAN, markers of RTEC senescence (p21, p16, SA-β-gal) positively correlate with renal interstitial injury [59]. Hypertensive nephropathy is associated with the susceptibility of vascular and interstitial cells to senescence [60]. In renal transplantation, it has been observed that older donors are more prone to ischemia-reperfusion injury (IRI) compared to their younger counterparts.

RTECs are particularly susceptible to cellular senescence when exposed to nephrotoxic drugs, ischemia-reperfusion injury, and other stressors associated with AKI. In addition to RTECs, podocytes, endothelial cells, immune cells, and interstitial cells also undergo stress-induced senescence [61]. Cellular senescence in AKI inhibits renal regeneration and repair, ultimately resulting in a poor prognosis. The downregulation of anti-senescence genes, such as klotho, and telomeres promotes cellular senescence in AKI and IRI mouse models [62, 63]. The key pathways driving cellular senescence in AKI are the p53/p21CIP1 and p16INK4a/Rb pathways. These pathways sequentially inhibit CDK complex formation and Rb phosphorylation, thereby suppressing E2F activity via Rb. In mild or moderate AKI, senescent cells are effectively cleared by immune cells, thereby promoting adaptive kidney repair. However, severe or persistent AKI leads to the accumulation of senescent cells that secrete the SASP through autocrine or paracrine mechanisms, causing inflammation, fibroblast proliferation, and extracellular matrix deposition. This culminates in maladaptive renal repair and fibrosis [64].

#### Others

Cellular senescence is also involved in various other medical conditions, including atherosclerosis, skeletal disorders, metabolic syndrome, idiopathic pulmonary fibrosis, and glaucoma. Senescent smooth muscle cells, for instance, secrete IL-6 and IL-8, which in turn promote the proliferation and migration of adjacent smooth muscle cells, ultimately leading to pulmonary arterial hypertension [65]. As individuals age, most cells in the bone microenvironment become senescent, displaying a heterogeneous SASP in both humans and mice. This phenomenon actively fosters the development of conditions such as osteoporosis and osteoarthritis [66, 67]. Furthermore, senescent cells play a crucial role in the exhaustion of stem cells and the senescence of macrophages, B lymphocytes, and T lymphocytes.

### Mechanisms of cellular senescence

Cellular senescence encompasses several mechanisms, including telomere damage or shortening, DNA damage, aberrant epigenetic modifications, mitochondrial dysfunction, nutrient-sensing imbalances, stem cell exhaustion, and disrupted cell communication. Recent studies have shown that chronic inflammation and ecological imbalance are correlated with cellular senescence [68].

#### DNA damage

The DNA damage response (DDR) is a crucial mechanism that triggers cellular senescence. DDR is activated in response to DNA damage and serves as a self-protective mechanism. However, sustained activation of DDR can promote cell-cycle arrest, ultimately resulting in cellular senescence. DNA damage leads to an increase in the phosphorylation of histone H2AX (γH2AX). This phosphorylation, in turn, activates the cell-cycle protein kinase inhibitors p16ink4a and p21cip1 through the p53 pathway. These proteins subsequently mediate the phosphorylation of the retinoblastoma tumor suppressor protein (Rb), culminating in cell-cycle arrest and the induction of cellular senescence [69].

#### Telomere shortening/damage

Telomeres are essential components that stabilize the ends of eukaryotic chromosomes, safeguarding the integrity of genetic information. These telomeres consist of a highly conserved non-coding repetitive DNA sequence, TTAGGG. Both telomerase and the shelterin complex play crucial roles in maintaining telomere structure and function [70–72]. Telomerase is responsible for maintaining telomeres and fulfilling a protective role. In mice, the loss of telomerase leads to premature senescence, while pharmacological activation of telomerase delays the aging process in aging mice [73]. Telomeres gradually shorten with each cell division, thereby promoting genomic instability and contributing to cellular senescence as well as senescence-related diseases. Telomere shortening represents the primary cause of replicative senescence, a phenomenon common to cellular senescence in various species, including humans and mice. Replicative senescence is observed in a multitude of cell types such as fibroblasts, keratinocytes, endothelial cells, lymphocytes, adrenal cortex cells, and chondrocytes. When telomeres shorten to a critical length, a sustained DNA damage response (DDR) is activated, ultimately leading to cellular senescence. Additionally, cellular stress-induced senescence, caused by various harmful stressors, leads to telomere shorting-induced DDR, ultimately resulting in cellular senescence.

#### Mitochondrial autophagy deficiency

Autophagy is a highly conserved process in eukaryotes, serving as the fundamental mechanism for the degradation and recycling of cellular components. Cellular autophagy is crucial for maintaining cellular metabolism, homeostasis, and the quality control of organelles [74]. Previous studies have revealed that levels of autophagy-related genes decrease with senescence, whereas increased autophagy has been shown to delay the senescence process [75, 76]. Mitochondrial autophagy plays a pivotal role in maintaining mitochondrial integrity. Defects in this process in the accumulation of damaged and fragmented mitochondria, leading to elevated production of reactive oxygen species (ROS). In contrast to non-specific clearance, mitochondrial autophagy selectively removes damaged or dysfunctional mitochondria through lysosomal degradation, ensuring mitochondrial quality. Research has demonstrated that mitochondrial autophagy is involved in various physiological and pathological processes, including early embryonic development, cell differentiation, and cell apoptosis [77]. In the kidney, which ranks second only to the heart in terms of mitochondrial abundance, mitochondrial damage is a key mechanism underlying kidney disease. Elevated ROS levels caused by mitochondrial damage are particularly crucial in cases of AKI [78]. Mitochondrial autophagy plays a protective role in damaged kidney tissues, especially in IRI [79, 80]. Activated mitochondrial autophagy effectively removes damaged mitochondria, subsequently reducing inflammation and ROS levels in in vivo and in vitro AKI models, thereby exerting a protective effect [81].

Mitochondrial autophagy is closely associated with cellular senescence. Loss of cellular autophagy leads to mitochondrial dysfunction and the accumulation of ROS, both of which are typical markers of senescence [82]. Numerous studies have demonstrated that mitochondrial dysfunction can induce cellular senescence [83]. Mitochondrial autophagy is essential for the development of senescence phenotypes [84]. Various stressors can trigger deficiencies in mitochondrial autophagy, ultimately promoting cellular senescence. Restoring autophagic function may delay the appearance of senescent phenotypes [85]. In senescent cells, the mitochondria exhibit a reduced mitochondrial membrane potential, decreased oxidative phosphorylation, and elevated ROS production [86–88]. Furthermore, while normal cellular mitochondria undergo a cycle of fission and fusion, mitochondria in senescent cells undergo excessive fusion. This may be associated with the downregulation of dynamin-related protein 1 (DRP1) and mitochondrial fission protein 1 (FIS1), which inhibit mitochondrial division and autophagy [89, 90]. Currently, two main pathways are involved in mitochondrial autophagy: PARKIN/PINK1-mediated mitochondrial autophagy and PARKIN/PINK1-independent receptor-mediated mitochondrial autophagy. The PINK1/Parkin-mediated pathway plays a protective role in AKI [91, 92]. Moreover, damage to PINK1-parkin-mediated mitochondrial autophagy has been linked to lung cell senescence, while restoring mitochondrial autophagy can delay cellular senescence [91]. In a mouse model of DN induced by high glucose levels, the absence of the autophagy receptor optneurin (OPTN) accelerated the senescence of RTEC [57]. Thus, mitochondrial autophagy is indispensable in the context of cellular senescence.

#### Cell metabolism

While senescent cells may lack proliferative capabilities, their metabolism remains highly active, characterized by an increased ratio of AMP/ATP to ADP/ATP ratio, enhanced glycolysis, and abnormal lipid metabolism [93]. The increase in the metabolic demand of senescent cells can be attributed to factors such as increased cell volume, increased SASP production, elevated ROS levels, and endoplasmic reticulum stress [94]. Both replicative senescence and stress-induced senescence exhibit enhanced glycolysis [95, 96]. Metabolic abnormalities involving malic acid, pyruvic acid, and fatty acids are implicated in cellular senescence [95]. Enhanced glycolysis is particularly necessary in radiation-induced senescence [97]. Disorders in lipid metabolism disorders and lipotoxicity contribute to cellular senescence and the establishment of a senescent microenvironment. Additionally, cellular metabolism regulates DNA damage and repair by balancing the nucleotide pool [98]. Key metabolic pathways, including the tricarboxylic acid (TCA) cycle, glycolysis, fatty acid (FA) synthesis, and nucleotide synthesis, are integral in maintaining cellular homeostasis and promoting DNA repair. Metabolic alterations exert a significant influence on the DDR and DNA repair processes. As a result, cellular metabolism is closely associated with senescence. Moreover, p53 can regulate cellular metabolism by inhibiting glucose uptake and glycolysis, while promoting the TCA cycle, oxidative phosphorylation, and fatty acid oxidation [7, 99]. Another crucial participant in cellular cycle arrest, RB, regulates the production of deoxyribonucleotides by controlling key enzymes through the transcription factor E2F1 [100].

#### Endoplasmic reticulum stress

Senescence is a persistent pathological process. At the molecular level, senescent cells accumulate abnormal DNA and proteins associated with the endoplasmic reticulum (ER) [96]. Elevated ROS levels trigger ER stress, leading to an imbalance in ER homeostasis. This imbalance results in the accumulation of unfolded proteins within the ER, prompting the activation of the unfolded protein response (UPR) [101]. ER stress can determine cell state or fate, including autophagy, apoptosis, or senescence [102]. A strong correlation exists between ER dysfunction and senescence, as senescent cells exhibit increased UPR [103–106]. Overactivation of ER stress may induce senescence and the SASP [101].

The UPR signaling pathway is activated in response to disruptions in protein homeostasis within the ER and plays a role in various kidney diseases [107]. Previous studies have demonstrated a correlation between ER stress and renal fibrosis [108]. ER stress marker glucose-regulated protein 78 (GRP78) is elevated in patients with DN and shows a positive correlation with senescent markers such as SA-β-gal and p21. This further confirms that advanced glycation end products promote the premature senescence of RTEC by activating ER stress-dependent p21 signaling. However, ER stress inhibitors can suppress premature senescence in RTEC and mitigate renal fibrosis [109, 110].

#### Epigenetic regulation

Epigenetics is the study of non-genotoxic, reversible, and heritable modifications that influence gene expression without altering DNA sequences [111]. Epigenetic remodeling is an important driver in cellular senescence and senescence-related diseases [112, 113]. Senescence is characterized by changes in gene expression, primarily regulated by dynamic epigenetic mechanisms. Senescent cells often exhibit chromatin remodeling, post-translational modifications of histones (PTMs), DNA methylation, and altered expression of non-coding RNAs [114–116]. To uncover common features of histone epigenetic modifications, chromatin spatial changes, and the significant role of the epigenetic factor KDM4 in senescent cells, researchers have utilized high-throughput epigenetic techniques such as quantitative proteomics, chromatin accessibility sequencing (ATAC-seq), and chromatin immunoprecipitation sequencing (ChIP-seq) [117].

DNA methylation (DNAm) is a relatively stable modification catalyzed by DNA methyltransferase (DNMT) that occurs in cytosine-guanine dinucleotide regions, known as CpG islands, and can be inherited by offspring DNA during replication. DNAm is a crucial epigenetic regulator of cellular senescence [118]. During cellular senescence, DNA methylation levels decrease, serving as a marker of cell senescence. DNA methylation influences gene expression and plays a regulatory role in senescence. For example, high methylation of the anti-senescence factor klotho promoter leads to decreased klotho gene expression, promoting the progression of CKD [119, 120]. DNA methylation is influenced by both exogenous and endogenous factors associated with cellular senescence. Nutritional factors, such as diets lacking folic acid and methyl donors, can lead to DNA hypomethylation. Dnmt1, a DNA methyltransferase, gradually decreases in activity during senescence, which has been associated with replicative senescence of smooth muscle cells and atherosclerosis. In ischemia-reperfusion AKI, Dnmt plays a central role in promoting renal fibrosis and inflammation [121].

Protein translational modifications (PTMs), including acetylation, phosphorylation, succinylation, butyrylation, SUMOylation, and lactylation, play essential roles in regulating protein function and gene transcription, and are closely associated with the epigenetic regulation of senescence and related diseases [122]. PTMs exhibit heterogeneity among different types of senescent cells. A comprehensive proteomic profiling study has revealed that approximately 700 cytoplasmic and nuclear proteins undergo post-translational modifications in senescent human cells [117]. Histone modifications occur in specific gene regions, such as histone H3 lysine, during cellular senescence [123]. In senescent endothelial cells, the levels of histone H3 lysine 4 trimethylation (H3K4me3) and H3K4 methyltransferase Smyd3 increase, promoting the production of the SASP [124]. Different regions of the tau protein have shown phosphorylation and ubiquitination modifications in Alzheimer’s disease [125], with phosphorylation sites being associated with pathological damage. SUMOylation has been linked to neurodegeneration [126]. In senescent kidney disease models, a close relationship between renal function and epigenetics has been observed [127].

## Role and mechanisms of RTEC senescence in AKI

RTECs constitute the most abundant cell population in the kidney, accounting for approximately 90% of the renal cortex. RTECs are the most vulnerable cells to hypoxia, proteinuria, toxins, and metabolic disorders, among others [61]. These cells are particularly abundant in mitochondria, especially within the S3 segment of proximal tubules. This abundance of mitochondria makes them highly vulnerable to injuries such as ischemia, hypoxia, and exposure to nephrotoxic drugs. These insults can lead to pathological responses, including cell necrosis, apoptosis, and senescence. Notably, the residual RTECs have an intrinsic repair capacity. They can re-enter the cell cycle from the G0 or G1 phase, undergo mitosis, and regenerate to form new RTECs. This regenerative capability plays a pivotal role in restoring kidney structure and function, ultimately determining the prognosis of renal health [128, 129]. Therefore, RTECs are not only the main instigators of AKI but are also critical in driving kidney repair and influencing the overall prognosis [128].

### RTEC senescence

RTECs contain numerous mitochondria that are prone to stress-induced senescence, particularly under certain conditions, such as ischemia and hypoxia [130]. These cells are highly sensitive to oxidative stress, mitochondrial dysfunction, and abnormal energy metabolism following AKI. These factors collectively contribute to cellular senescence and hinder the proliferation of surviving RTECs, thereby inhibiting renal regeneration [131]. In the context of AKI, the binding of CDK with cyclin forms a cyclin-CDK complex that triggers cell-cycle arrest at the G1/S checkpoint, culminating in cellular senescence. Notably, in animal models of AKI caused by various factors, such as ischemia-reperfusion, folic acid, cisplatin, aristolochic acid, and contrast agents, a large number of p16- and p21-positive senescent RTECs has been observed during the early stages of AKI. The accumulation of senescent RTECs is closely associated with interstitial fibrosis and renal dysfunction. Furthermore, the secretion of proinflammatory cytokines such as IL-6 and IL-8 increases after AKI [132, 133]. Single-cell transcriptomic analysis has revealed that Vcam1 + /Ccl2+ RTECs at a late injury stage (28 days) can be distinguished by the activation of NF-κB, TNF, and AP-1 signaling pathways. These cells exhibit secretory phenotypes typical of senescent cells [134]. These findings indicate that senescent RTECs not only inhibit tubular regeneration and repair but also contribute to the progression of AKI to CKD through the SASP. Additionally, senescent cells have the ability to induce senescence in surrounding normal cells via paracrine signaling pathways, thereby significantly impacting the poor prognosis associated with AKI [135, 136].

Numerous studies have shown that RTEC senescence is primarily dependent on the P16Ink4a/Rb and/or P19Arf/P53/P21Cip1 pathway following AKI. Recent studies have uncovered the role of the Wnt9/β-catenin signaling pathway in regulating P16 and P53/P21, leading to cell-cycle arrest and senescence in IRI. The SASP, in turn, promotes fibroblast proliferation and extracellular matrix protein deposition, ultimately culminating in renal fibrosis following AKI [133]. In UIRI mice, BRG1 inhibits autophagy to induce RTEC senescence through the Wnt/β-catenin signaling pathway, ultimately contributing to renal fibrosis [137]. The ectopic expression of Klotho significantly delays fibrotic lesions and activates the Wnt/β-catenin signaling pathway by inhibiting cell senescence [138]. Myd88, a key molecule in the Toll-like receptor/interleukin (IL)-1 receptor signaling, regulates RTEC senescence via Toll-like receptor signaling [133]. Increasing evidence suggests that mitochondrial dysfunction induces cell senescence, and senescent cells accumulate numerous dysfunctional mitochondria. Mitochondrial dysfunction exacerbates RTEC senescence by activating ROS and disturbing energy metabolism. Moreover, SIRT1, vascular non-inflammatory molecule 1 (Vanin-1, VNN1), and other factors are closely associated with RTEC senescence in AKI, implying that RTEC senescence is the result of multiple biological mechanisms working in concert following AKI.

## Intervening RTEC senescence in AKI

### Inhibiting RTEC senescence

**Fig. 2:**
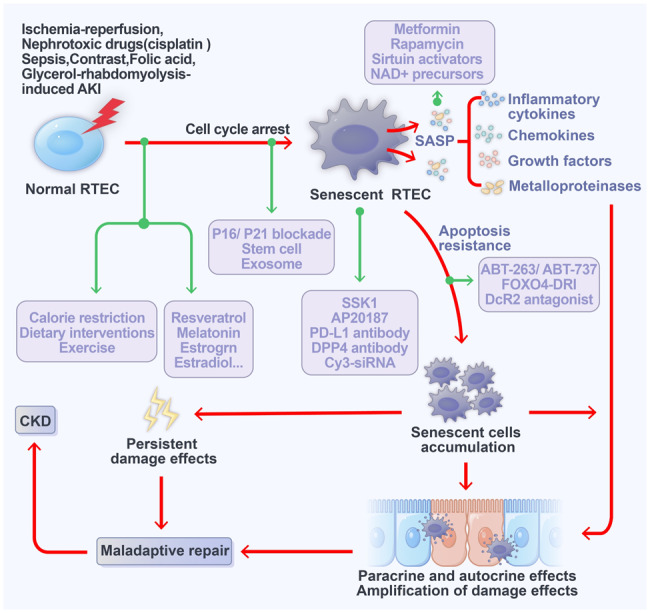
Senescence interventions. Inhibiting cell senescence, targeted clearance of senescent cells, promoting apoptosis of senescent cells, and inhibiting SASP release could be potential methods for improving renal prognosis following AKI.

Cellular senescence is a crucial event in the progression of AKI, and several strategies have been explored to delay or mitigate cellular senescence (Fig. 2), including calorie restriction and increased exercise. These methods extend the lifespan and reduce oxidative stress levels and age-related inflammation [139, 140]. Dietary interventions, including the consumption of nutrients such as betaine, choline, and folic acid, can influence age-related diseases by modulating epigenetic processes and the gut microbiota. Conversely, excessive glucose intake has been observed to reduce microbial diversity in animal models, contributing to the advancement of age-related diseases [141]. Furthermore, the quality of sleep and regular exercise have been shown to influence senescence and the levels of SASP [142]. Strategies to neutralize SASP have become vital for inhibiting senescence, encompassing the inhibition of signal cascades associated with SASP in senescent cells, interference with SASP secretion, or inhibition of the components comprising the SASP [143]. Several anti-senescence drugs (Fig. 2), including metformin, rapamycin, sirtuin activators, and NAD+ precursors, have been developed in animal models. Metformin and rapamycin, for instance, activate autophagy, improve mitochondrial function, regulate SASP secretion, inhibit mTOR, and contribute to lifespan extension [144, 145]. Resveratrol activates SIRT, thereby exerting antioxidant and anti-inflammatory effects. Other substances, such as melatonin, estrogen, and estradiol, are known to inhibit SASP production and regulate the release of inflammatory factors [146–148]. Recent studies have unveiled the anti-senescence properties of stem cells and their extracellular vesicles, with potential applications in retarding cellular senescence and the progression of senescence-related diseases. Multiple studies have explored these effects in the context of heart, blood vessels, cartilage, skin, and kidney damage. Furthermore, interventions targeting hypertension and the renin-angiotensin-aldosterone system have been found to reduce the number of senescent cells, thereby delaying renal senescence.

When considering the mechanisms of cell senescence in AKI, one potential intervention target is cell-cycle arrest (Table 1). For example, in IRI models, p21 knockout has been shown to promote the proliferation of RTECs, but it had no discernible effect on renal function or mortality rates [149]. In a cisplatin-induced AKI model, p21 gene knockout led to increased necrosis and apoptosis of RTECs and exacerbated renal tissue damage [150]. Conversely, p16 gene knockout has been found to promote RTEC proliferation and facilitate the recovery of renal function after AKI, thereby ameliorating renal tubular atrophy, interstitial fibrosis, and collagen deposition [151, 152]. Furthermore, Smad7 has been shown to improve G1 cell-cycle arrest via the TGF-β/Smad3-p21/p27 pathway, thus inhibiting AKI progression [153]. Mesenchymal stem cells (MSCs) and their extracellular vesicles, exosomes, also play protective roles in mitigating RTEC senescence. For instance, human umbilical cord-derived MSCs have been demonstrated to protect rat kidneys from senescence induced by AKI [154]. Moreover, Klotho gene-modified bone marrow-derived MSCs promote RTEC proliferation and repair by inhibiting the Wnt/β-catenin pathway in AKI [155]. Extracellular vesicles containing miRNA let-7b-5p have been found to alleviate RTEC senescence by inhibiting p53, thereby delaying the progression of AKI to CKD [156]. G protein-coupled receptor (GPCR) family member free fatty acid receptor 4 (FFAR4) is a potential drug target against AKI. The expression of FFAR4 was abnormally decreased in RTECs of cisplatin, CLP, and IRI-induced AKI. Activation of FFAR4 by agonist TUG891 alleviated AKI by regulating cellular senescence [157]. A ligand-activated transcription factor aryl hydrocarbon receptor (AhR) was abnormally increased in the kidneys of cisplatin-induced AKI mice. Notably, inhibition of AhR could improve cellular senescence of injured kidneys, which was indicated by SA-β-gal activity, biomarker p53, p21, p16 expression, and SASP [158].

**Table 1:** Effects of inhibiting cell senescence on renal outcomes in AKI models.

Animals	Renal model	Mechanism of inhibiting cell senescence	Evidence of inhibiting cell senescence	Renal outcome	Reference
p16 KO mice	UUO	Impaired cell-cycle arrest	SA-β-gal positivity ↓ SASP (IL-6, TGF-β) ↑ PCNA ↑	Interstitial fibrosis ↑ Blood urea nitrogen ↑	[159]
p16 KO mice	Renal transplant	Impaired cell-cycle arrest	Ki-67 ↑	Interstitial fibrosis and tubular Atrophy ↓ Creatinine clearances ↑ Renal survival ↑	[151]
p16 KO mice	Glycerol-rhabdomyolysis-induced AKI	Impaired cell-cycle arrest Inhibiting NF-κB Downregulating ROS signaling	SASP (IL-1β, IL-6, TNF-a) ↓ ROS ↑	Renal damage ↓ Serum urea ↓ Serum creatinine ↓	[152]
p16 /p19 double KO mice	Renal IRI	Impaired cell-cycle arrest	p16 ↓ p19 ↓ Ki-67 ↑	Kidney regeneration ↑ Microvascular repair ↑ Serum creatinine ↓	[132]
p21 KO mice	Renal IRI	Impaired cell-cycle arrest	PCNA ↑	Mortality ↑ Blood urea nitrogen ↑	[149]
p21 KO mice	Cisplatin-induced AKI	Impaired cell-cycle arrest	Brdu ↑ PCNA ↑	Renal damage ↑ Blood urea nitrogen ↑ Mortality ↑	[150]
p21 KO mice	Renal IRI	Impaired transient cell-cycle arrest	Ki-67↑	Renal survival ↓ Blood urea nitrogen ↑ Renal damage and fibrosis ↑	[160]
	Renal ischemic-preconditioned IRI	Impaired transient cell-cycle arrest	Ki-67↓ MCP-1, IL-6↑ Oxidative stress↑		

### Targeted clearance of senescent RTECs

The targeted clearance of senescent cells represents a significant advancement in the prevention and treatment of age-related diseases (Fig. 2) [7]. Current methods for the targeted clearance of senescent cells primarily encompass transgenic approaches, the use of specific drugs or interference peptides, and immune cell-targeted clearance. β-gal is widely recognized as a biomarker for cellular senescence and is often the focus of drug-based interventions. SSK1 is specifically activated by β-galactosidase, enabling the targeted elimination of senescent cells. This highlights the utility of lysosomal β-gal as an effective tool for the selective removal of senescent cells [159]. Programmed cell death ligand 1 (PD-L1) is expressed and accumulates in senescent cells and is closely linked to the SASP. The use of PD-1 antibodies has been shown to reduce the number of p16-positive cells in the body and improve senescence-related phenotypes [160]. A variety of strategies for the targeted clearance of senescent cells is presented in Table 2. In a mouse model of premature senescence, which presented numerous p16-positive senescent cells in the kidneys and heart, transgenic technology facilitated the selective elimination of p16-positive senescent cells using AP20187, which targets the senescence marker p16. This approach significantly improves kidney fibrosis and myocardial hypertrophy, ultimately extending the lifespan of the mice [161, 162]. In a mouse model of chronic ischemic renal injury, intraperitoneal injection of AP20187 significantly inhibited renal interstitial fibrosis, improved renal function, and increased renal tissue oxygen content by specifically targeting the clearance of p16-positive senescent cells [163]. These findings indicate that the targeted clearance of p16-positive senescent cells not only alleviates kidney damage and enhances renal function but also has protective effects on other organs. Furthermore, ganciclovir has been found to induce apoptosis in p16-positive senescent cells in p16-3MR transgenic mice, resulting in improved renal fibrosis and function, and the inhibition of SASP production [161]. However, it is important to note that p16 is not a specific marker for senescent RTECs. p16 is also expressed in all senescent cells within the renal parenchyma, including vascular endothelial cells, podocytes, and interstitial fibroblasts. Targeted clearance of p16-positive senescent cells may lead to structural damage and renal dysfunction. Previous studies have utilized proteomic screening to identify the specific marker DPP4 on the membranes of senescent fibroblasts. This marker inhibits the immune clearance ability of NK cells. Treatment with a DPP4 antibody has been shown to promote the clearance of senescent NK cells [161, 164]. Furthermore, proximal RTECs are the primary site for renal uptake of siRNA. Following ischemic injury, intravenous administration of Cy3-siRNA rapidly delivers the substance to proximal RTECs, significantly reducing p53 expression. This approach effectively mitigates damage to tubular cells [165]. Nano Drug Delivery (Nano-DDS) represent a highly promising drug delivery strategy that has been extensively studied and documented. This nanotechnology enables efficient drug targeting to diseased tissues, resulting in reduced dosage, minimal damage to healthy cells, and enhanced drug bioavailability. Nanotechnology offers a new therapeutic approach for kidney diseases, including AKI [166]. Research has shown that medium-sized nanoparticles (400 nm) can selectively target RTECs [167]. Nanotechnology enables the sustained or controlled release of drugs within the body. Importantly, the kidney exhibits 26–94 times higher uptake efficiency of mid-sized nanoparticles compared to other organs [168]. Thus, nanotechnology holds promise as a novel approach for the targeted removal of senescent RETCs. These studies suggest that the targeted elimination of senescent cells has the potential to serve as a new strategy for preventing and treating the progression of kidney diseases.

**Table 2:** Effects of targeted clearance of senescent cell on renal outcomes in renal models.

Animals	Renal model	Methods for clearing senescent cell	Evidence of senescent cell clearance	Renal outcome	Reference
INK-ATTAC mice	Natural aging	AP20187 administration to deplete p16- positive cells	SA-β-gal positivity ↓	Glomerulosclerosis ↓ Blood urea nitrogen levels ↓	[50]
INK-ATTAC mice	Renal artery stenosis-induced ischemic Nephropathy	AP20187 administration to deplete p16- positive cells	SA-β-gal positivity ↓ P16 positivity ↓ SASP (IL1a, IL6, Ccl2, Mmp3, TNF-a) ↓	Interstitial fibrosis ↓ Renal perfusion ↑	[163]
Xpd^TTD/TTD^ Mice	Fast-aging	FOXO4-DRI induces targeted apoptosis of senescent cells	Lamin B1 ↑ SASP (IL1a, IL1β, IL6) ↓	Serum urea ↓ Serum creatinine ↓	[170]
p16-3MR	Natural-aging	Ganciclovir induces p16-positive-restricted cell apoptosis	Lamin B1 ↑ SASP (IL6) ↓	Serum urea ↓ Serum creatinine ↓	[170]
p16-3MR	Folic acid-induced AKI	Ganciclovir induces p16-positive senescent cell apoptosis	SA-β-gal positivity ↓ Lamin B1↑ Ki-67 ↑	Interstitial fibrosis ↓ No effects on tubular damage	[133]
Wild-type mice (C57BL/6J)	Folic acid-induced AKI	FOXO4-DRI induces apoptosis of senescent cells	SA-β-gal positivity ↓ Lamin B1↑ Ki-67 ↑	No effects on kidney damage and interstitial fibrosis	[133]

### Promoting senescent RTECs apoptosis

Resistance to apoptosis is one of the main characteristics of senescent cells. Recent studies have revealed that Bcl-2/Bcl-xL specific inhibitors (Fig. 2), such as ABT-263/ABT-737, can selectively induce apoptosis in senescent cells, thereby contributing to the rejuvenation of stem cells in aged tissues [22, 169]. ABT-263 has progressed to clinical trials for the treatment of hematological malignancies and solid organ tumors. The synthesis of a small-molecule interference peptide known as FOXO4-DRI is aimed at disrupting the interaction between FOXO4 and the p53 apoptotic signaling pathway. This selective induction of apoptosis in senescent cells by FOXO4-DRI has shown promise in improving renal function, hair density, and the maintenance of tissue and organ homeostasis [170]. However, it is important to note that in the early stages of FA-induced AKI, administering FOXO4-DRI selectively induces senescence in RTECs, thereby improving renal inflammation and interstitial fibrosis [133]. Recent studies have found that senescent RTECs and fibroblasts express the decoy receptor DcR2, which is closely associated with the resistance to apoptosis observed in senescent cells [171]. Elevated DcR2 expression in fibroblasts during senescence serves to antagonize immune cell clearance. Conversely, downregulation of DcR2 expression promotes NK cell clearance of senescent cells, thereby inhibiting liver fibrosis [171]. In the context of senescent RTECs, inhibiting DcR2 expression has been observed to promote apoptosis, reduce SASP secretion, and inhibit renal interstitial fibrosis [24]. These findings suggest that promoting apoptosis in senescent RTEC may represent an effective approach for improving AKI prognosis.

## Summary

RTEC senescence is a key cellular event in the progression of AKI. It not only hinders the proliferation and regeneration of RTEC but also contributes to the release of SASP, which promotes inflammation and fibrosis. This can be referred to as the “Quagmire effect”. The unique SASP and anti-apoptotic capabilities of senescent cells make them promote the transformation of surrounding cells into senescent cells. Senescent cells continuously accumulate, forming something akin to a quagmire, and continually dragging normal cells to expand their size. As a result, a large number of senescent cells can lead to the occurrence of chronic inflammation, with some chronic inflammation spreading to the entire kidney, ultimately leading to kidney dysfunction.

Currently, strategies aimed at inhibiting RTEC senescence, clearing senescent RTECs, and promoting apoptosis of senescent cells show promise in improving AKI prognosis. These approaches hold substantial potential for application in clinical settings. However, the challenge of achieving precise and targeted intervention for RTEC senescence to enhance renal outcomes in AKI remains an unresolved issue. Further exploration of senescent cell-specific biomarkers and underlying mechanisms may yield novel insights and strategies to promote the regeneration and repair of AKI.
